# Overview of Systematic Monitoring Networks for Surface Water Quality in the Amazon River Basin

**DOI:** 10.1007/s00267-026-02546-6

**Published:** 2026-06-25

**Authors:** Luanna Costa Dias, Luiza Carla Girard Mendes Teixeira, Lindemberg Lima Fernandes, João Batista Marcelo de Lima, Hugo de Souza Ferreira, Vinicius Silva de Oliveira

**Affiliations:** 1 Federal University of Pará, PPGEC/ITEC/UFPA, Belém, PA Brazil; 2 Geological Survey of Brazil, SGB, Belém, PA Brazil

**Keywords:** Monitoring point, Historical series, Systematic monitoring, Amazon, Water management

## Abstract

Water quality monitoring is essential for water resources management, especially when conducted systematically (continuous, standardized, and with data available to users), as it generates historical time series for each observed variable. The Amazon River basin plays a crucial role in biodiversity conservation and climate regulation, in addition to supporting a wide range of economic activities, making systematic monitoring extremely valuable for decision-making. Therefore, the objective of this study is to evaluate the systematic water quality monitoring landscape in this basin. This research identified five networks operating in the region: the National Hydrometeorological Network (RHN), the Surface Water Quality Monitoring Network (RNQA), the virtual Hidrosat network, the Amazon Water, Air, and Soil Quality Monitoring Program (ProQAS/AM), and the international So Hybam Observatory.The RHN is the oldest network and has the best spatial distribution; however, it measures only basic parameters and presents temporal gaps. Hidrosat exclusively estimates suspended sediment concentration through satellite data. ProQAS/AM operates systematically in the region of Manaus (Amazonas State, Brazil), providing data for the Water Quality Index (WQI) variables of the Negro River. The RNQA is a recent initiative that includes microbiological data, established by the Brazilian National Water and Sanitation Agency (ANA) and coordinated by Brazilian states to generate continuous information; however, its stations are spatially concentrated, leaving geographic gaps. Finally, the So Hybam Observatory stands out as an international cooperation initiative focused on transboundary rivers along the Amazon system from the Andes, measuring geochemical parameters and isotopes since 2003, albeit with restrictions in temporal frequency. This study demonstrates that each dataset has specific characteristics and faces monitoring gaps in the Amazon, highlighting the need for expansion and better standardization. Nevertheless, the integration of these networks enables the development of novel studies on temporal analysis and water body classification, which are fundamental for water resources management. Furthermore, the international landscape reveals that the most recent research focuses on system integration and sampling efficiency, positioning this article as a milestone for information unification in the region.

## Introduction

Surface water is the most important and accessible source of water for human life and agro-industrial production. However, because it is easy to collect, it is the most polluted in many countries (Aboutalebi et al. 2016; Jiang et al. 2020).

Given this, monitoring water quality is essential for water resource management, as it is important for understanding and protecting aquatic environments, elucidating the processes that affect water quality, and detecting and analyzing spatial and temporal trends.

The topic of design and optimization of water quality monitoring networks is frequently studied around the world. The first monitoring networks appeared in the 1960s (Sanders et al. 1983), but according to Chapman and Kimstach (1992), modern monitoring began in the 1950s with a focus solely on data collection. Sharp (1971) conducted one of the first studies in the literature to optimize water quality monitoring points, with the objective of locating sources of pollution.

For classic authors on this topic (Sharp 1971; Sanders et al. 1983; Chapman and Kimstach 1992; Harmancioglu et al. 1999), the definition of water quality monitoring is to obtain physical, chemical, and biological control of water characteristics using statistical sampling. These works highlight the complexity of monitoring, the need to optimize the network, and emphasize the particularities and difficulties for developing countries, as mentioned by Gradilla-Hernández et al. (2022): “in countries in the southern hemisphere, water quality monitoring networks are inefficient due to the use of subjective strategies and insufficient investment”.

According to Sharp (1971), the three stages of network design are: selection of indicator parameters, definition of sampling locations, and determination of collection frequency. In addition, the specific objectives of monitoring, easy access to sampling points, representativeness in the watershed, surveillance of pollution sources, estimation of pollutant loads, and water use must be considered (Aboutalebi et al. 2016).

Based on Destandau and Zaiter (2020), there are two types of networks: surveillance control networks and operational control networks (which are temporary). The surveillance control network is like the monitoring networks operated by government agencies to comply with their environmental legislation objectives. This type of network is also known as systematic monitoring, which, according to Libos et al. (2023), consists of fixed sampling points with defined parameters, methodologies, and frequencies. The operational control network, on the other hand, corresponds to the self-monitoring of potentially polluting companies, which is also called non-systematic monitoring, as it is not standardized and has a random frequency.

In Brazil, water monitoring networks were established based on state monitoring, without any standardization, with the first Brazilian networks beginning in the 1970s (ANA 2012). In 1974, CETESB (São Paulo State Environmental Company) began monitoring the water quality of rivers and reservoirs to control pollution, and adapted the Water Quality Index (WQI), which is widely used in Brazil (Medeiros 2012).

Until 2014, Brazil’s surface water quality monitoring networks were not standardized, with only 17 Brazilian states monitoring water quality systematically. It was only with the creation of the Surface Water Quality Monitoring Network (RNQA) by the National Water and Basic Sanitation Agency (ANA) in 2013 that guidelines for operation and systematic monitoring were established (ANA 2012).

In 2016, all states in the Northern Region of Brazil, with the exception of Tocantins, did not have a state monitoring network (ANA 2022), which means that the rivers in these states, most of which are part of the Amazon River basin, lack adequate knowledge of water quality in the world’s largest hydrographic basin.

Given this scenario, the objective of this study is to assess the current situation of surface water quality monitoring networks in the Amazon River basin through a spatiotemporal analysis of existing points, measured parameters, and data frequency. In addition, the situation in the Amazon is compared with other Brazilian and international networks. Thus, an overview of surface water quality monitoring in an area of global relevance is presented.

## Study Area

**Fig. 1:**
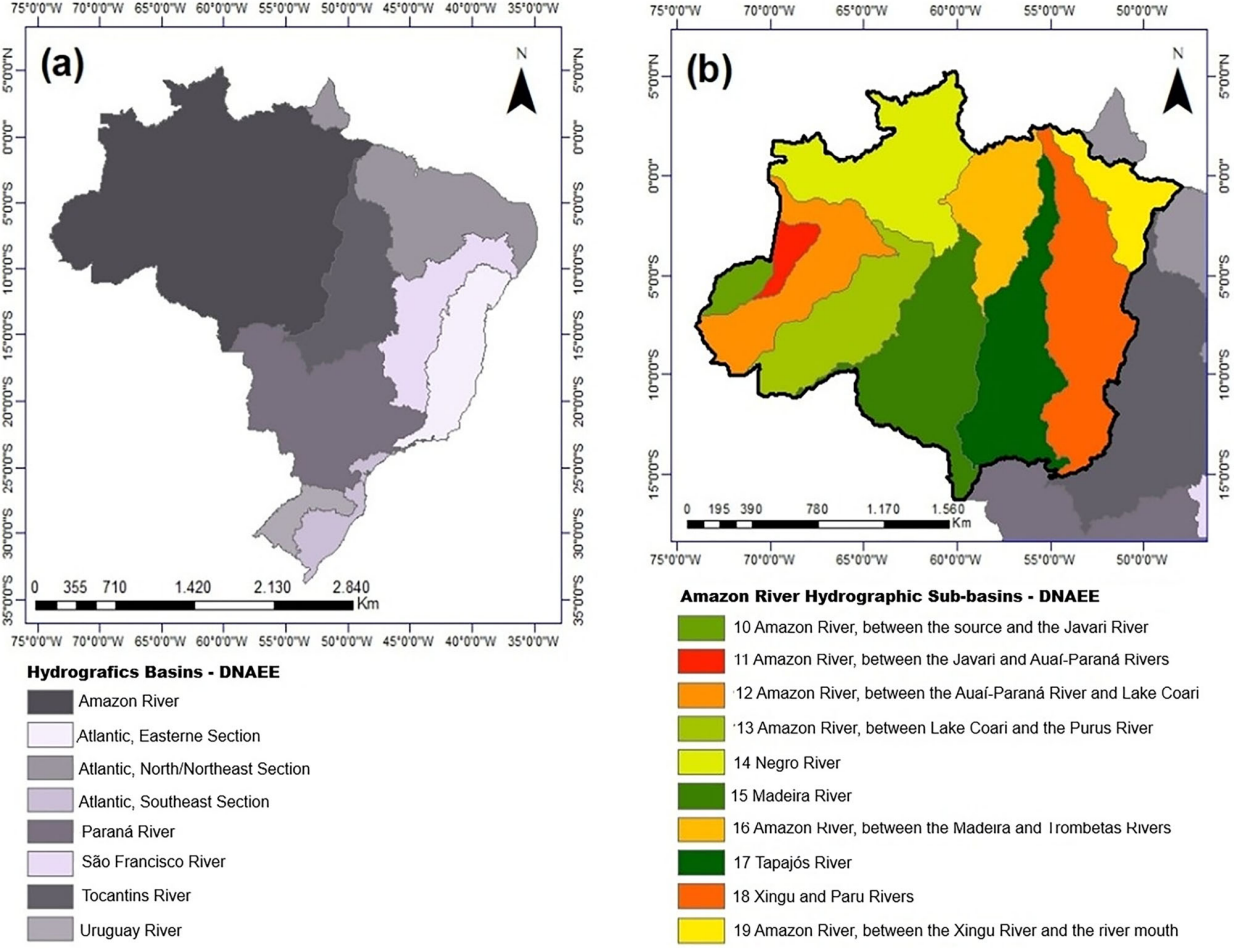
Division of the former National Department of Water and Electric Power (DNAEE) – Amazon River Basin (Brazilian portion). **a** Division of Hydrografics Basins in Brazil and **b** Division of Amazon River Hydrographic Sub-basins Source: Dias et al. (2022)

The division of Brazilian territory into river basins was carried out in 1972 by the former National Department of Water and Electric Power (DNAEE), with the aim of implementing an information system and coding the gauging stations that make up the National Hydrometeorological Network (RHN). In this division, there are nine river basins, each of which is subdivided into ten sub-basins, Fig. 1a. Basin 1 corresponds to the Amazon River, which is the study area, as shown in Fig. 1b.

The Amazon River basin covers ~6.3 million km² in Brazil and includes the states of Acre, Amazonas, Rondônia, and Roraima, as well as parts of the states of Amapá, Mato Grosso, and Pará (Dias et al. 2022). It has a population of ~12 million inhabitants, which corresponds to about 5% of Brazil’s population, with the most populous urban centers being Manaus (2 million inhabitants), Porto Velho (517,000 inhabitants), and Rio Branco (387,000 inhabitants) (IBGE 2023). This vast territorial expanse and low population density, which is concentrated in urban centers, hinders logistical access for obtaining conventional hydrological data across its entire area.

According to ANA (2012) and OTCA (2023), the main pressures on water quality in the Amazon basin are: domestic sewage and solid waste, industrial activities such as the Manaus Free Trade Zone, mining and artisanal mining in the Tapajós and Madeira basins, deforestation and inadequate land management (the deforestation arc in the southern part of the basin), hydroelectric development, navigation (which is the only means of transportation in many places), and the effects of droughts such as those of 2005, 2010, 2023, and 2024.

The natural conditions of the waters in the Amazon River basin are determined by the geology and vegetation that establish their physical and chemical characteristics (ANA 2012). In the Amazon basin, there are significant areas of sedimentation originating from the Andes (Brigel and Gutierrez 2024). The colors they take on were classified by Sioli (1984) using geological formation, watercolor, sediment load, electrical conductivity, and humus formation (organic matter), which is directly related to a decrease in pH. Based on this, they were classified as white waters (neutral pH between 6.2–7.2), black waters (low pH of 3.8–4.9), and clear waters (pH between the extremes of white and black waters, ranging from 4.5 to 7.8) (Rudorff et al. 2011).

White waters are highly turbid and muddy in color; they originate in the Andes and are rich in mineral salts and suspended matter, such as those of the Solimões, Madeira, Juruá, and Purus rivers. Black waters, on the other hand, are dark because they drain lowland areas and forests with soils rich in organic matter, such as the Rio Negro. Clear waters are greenish or transparent because they drain from crystalline areas such as ancient rocks, such as those of the Tapajós River (Sioli 1984; Brigel and Gutierrez 2024; Duvoisin Jr. et al. 2025).

The hydrological and hydrochemical conditions of Amazonian rivers pose a complex challenge for characterizing, standardizing, and monitoring water quality. Added to this is the magnitude of the flow, which exceeds 130,000 m³/s (ANA 2012; Coutinho et al. 2019), and the range of the annual rainfall, which varies from 2000 to 2500 mm/year (Fisch et al. 1998; Ishihara et al. 2014; Molinier et al. 1995), make the operation and maintenance of systematic water quality monitoring networks even more difficult, requiring efficient and tailored sampling strategies for this specific context.

## Materials and Methods

### Data Collection and Definition of the Systematic Network

To achieve the study’s objectives, we first conducted a survey of the information available at ANA, state government agencies, and public institutions (such as universities and environmental agencies) regarding the networks that monitor water quality.

The selection criteria used to classify a network as “systematic” were based on the studies by Destandau and Zaiter (2020) and Libos et al. (2023). To be selected, monitoring points had to simultaneously meet four quantitative and qualitative criteria: Spatialization: explicit geographic coordinates; Standardization: use of standardized analytical protocols; Temporal continuity: continuous sampling routines, rather than short-term project campaigns; and Public availability: data accessible via Geographic Information Systems (GIS) on a website.

**Fig. 2:**
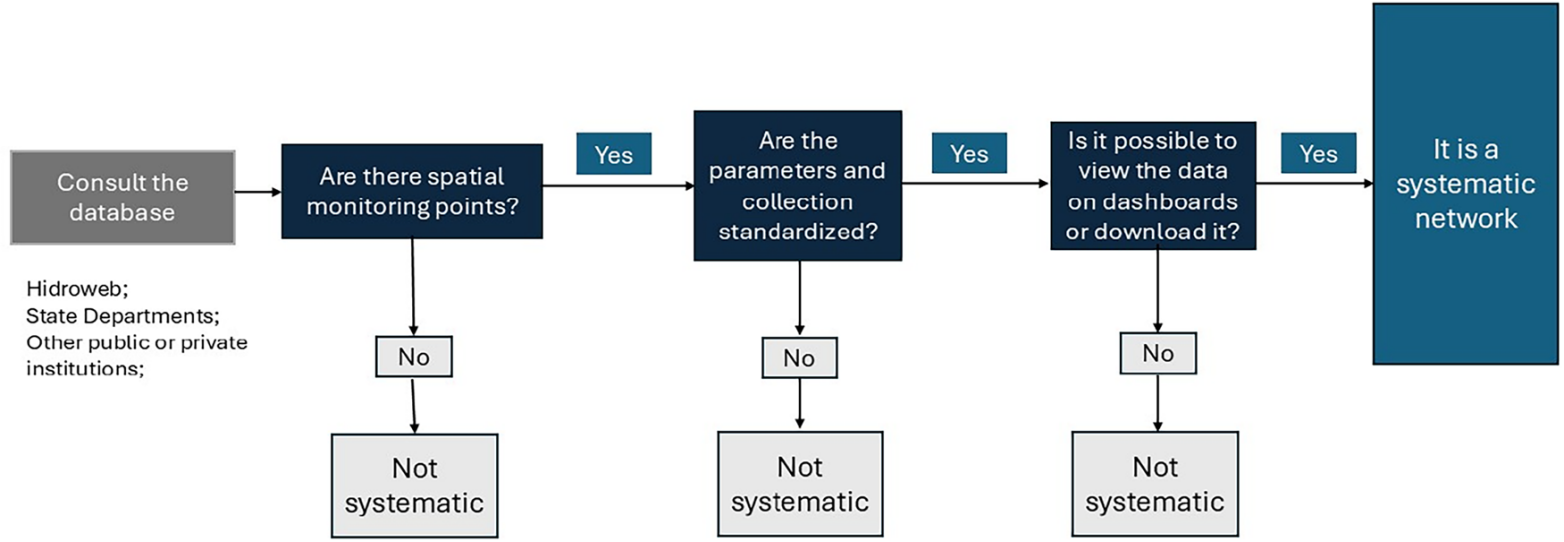
Flowchart for identifying systematic networks

Networks that met all four requirements were classified as systematic water quality monitoring networks (Destandau and Zaiter 2020). Figure 2 illustrates the operational flowchart for this stage.

### Data Acquisition and Compilation of Time Series

After identifying the monitoring networks, historical datasets up to the year 2024 were compiled within the boundaries of the Amazon River basin and its sub-basins (DNAEE classification, Fig. 1b). Data were acquired by downloading the raw time series directly from the official platforms of each managing institution.

To assess the consistency and completeness of the data, the historical series for each station was analyzed. The attributes analyzed included the number of monitored parameters, the operating entities, the sampling frequency, and the quantification of data gaps and errors. This step was essential to determine whether systematic monitoring provides consistent, long-term information capable of supporting trend analyses and characterization of the water body.

### Spatio-Temporal Analysis and Performance Evaluation

The geographic coordinates of each monitoring station were georeferenced using QGIS software (version 3.28) to analyze their spatial coverage within the study area.

To assess the adequacy of the spatial distribution, the network density was evaluated against the official standard established by ANA Resolution No. 903 (ANA 2013) for the National Surface Water Quality Monitoring Network (RNQA). For Region 1 (which covers the Amazon), the resolution establishes a minimum acceptable limit of one monitoring point per 10,000 km². The Spatial Density Index (DI) was calculated for each of the 10 sub-basins using Eq. (1):

1 $${DI}=\frac{{N}_{{stations}}}{{A}_{{sub}-{basin}}}\times 10,000$$

Where ${N}_{{stations}}$ is the number of active systematic stations in the sub-basin, and ${A}_{{sub}-{basin}}$ is the total area of the sub-basin (km²). Compliance with the regulatory minimum (DI ≥ 1.0) was verified for each sub-basin in order to identify areas of critical spatial gaps.

After presenting the results, the data are compared with those from other major Brazilian and international networks. In the case of Brazilian networks, those with the best monitoring performance—as defined by ANA (ANA 2013)—were selected. In the international context, the information was obtained from a review of scientific articles of significant global relevance, given the difficulty of obtaining official data from other countries.

## Results

Systematic monitoring of surface water quality in Brazil was conceived through state initiatives in which each state adopted its own criteria for monitoring. In the 1990s, with the advent of the National Water Resources Policy (BRAZIL 1997), which instituted decentralized and participatory management and has as one of its objectives “to ensure the availability of water at adequate quality standards,” the focus began to shift toward the creation of a national water quality monitoring network.

According to ANA (2012), water quality monitoring in Brazil began in the 1970s, through the measurement of some basic parameters at RHN fluviometric points and by environmental agengy, such as CETESB. According to Technical Note No. 62/2023/SGH from ANA (2023), the first specific water quality monitoring network began in 1990 with its implementation in the Rio Doce basin. From then on, a specific network was designed to monitor water quality, rather than relying on the RHN’s fluviometric stations.

The only initiative to improve these networks took place in 2008 with the launch of the National Water Quality Assessment Program (PNQA), whose main objective was to provide society with adequate knowledge about the quality of Brazilian surface waters. One of the components of the PNQA is the Surface Water Quality Monitoring Network (RNQA), which aims to expand and optimize the geographic distribution of water quality data (ANA 2023). The RNQA is published in ANA Resolution No. 903 (ANA 2013), and states participate on a voluntary basis.

**Fig. 3:**
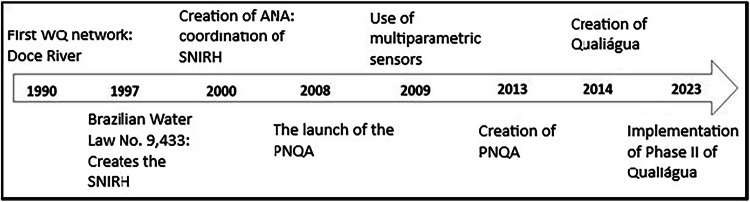
Temporal evolution of systematic water quality monitoring at the federal level of the Brazilian government

To encourage states and strengthen the RNQA, in 2014, through Resolution No. 1040, which was replaced by Resolution No. 643 (ANA 2016), the Program to Encourage the Disclosure of Water Quality Data (Qualiágua) was launched, with awards given to states and the Federal District for achieving goals in the implementation and operation of the RNQA. The program is divided into phases lasting 60 months, with the second phase being implemented in 2023 (ANA 2016). Figure 3 shows the timeline of these milestones in the creation of the RNQA, which is the materialization of the Brazilian federal government’s incentive for water quality monitoring.

Within the study area, which is the Amazon River Basin, there were no state water quality monitoring networks until the creation of the RNQA, except for Mato Grosso, which has had water quality data records kept by its state secretariat since 2006. The only systematic water quality data before the RNQA within the study area were those from the RHN. Thus, with the creation of the RNQA, there is now another systematic and specific network for water quality.

This study also presents the Hidrosat systematic network (Integrated System for the management, processing, and dissemination of hydrological data obtained by satellites), managed by ANA, which contains satellite-estimated water quality data with continuous serial records since 1985. Similarly, the research extends to the systematic network created at the University of the State of Amazonas (UEA), with a state-of-the-art laboratory structure that systematically monitors the main river basins in this study area through the creation and coordination of the Program for Monitoring Water, Air, and Soil Quality in the State of Amazonas (ProQAS/AM). Finally, there is the So Hybam (Amazon basin water resources observation service) network, an observatory created in 2003 to monitor rivers in the Amazon, which provides basic hydrological, sediment, and hydrogeochemical data. The following sections present and analyze each of these systematic networks.

### National Hydrometeorological Network

The RHN focuses on quantitative data, and monitoring guidelines have been in place since 1920 for studies on hydroelectric power use (DIAS 2022). The focus on multiple uses of water has developed over time.

The RHN data are available on the hidroweb (https://www.snirh.gov.br/hidroweb/serieshistoricas) platform of ANA and were consulted from the fluviometric stations. This research found water quality data from 30 stations dating from between 1975 and 1985. However, these series were interrupted in the late 1980s, and monitoring only resumed in the 2000s. Currently, measurement campaigns are conducted four times a year, according to a schedule established by the SGB for the validation of key curves. In this network, water quality data are secondary and dependent on streamflow measurements.

**Fig. 4:**
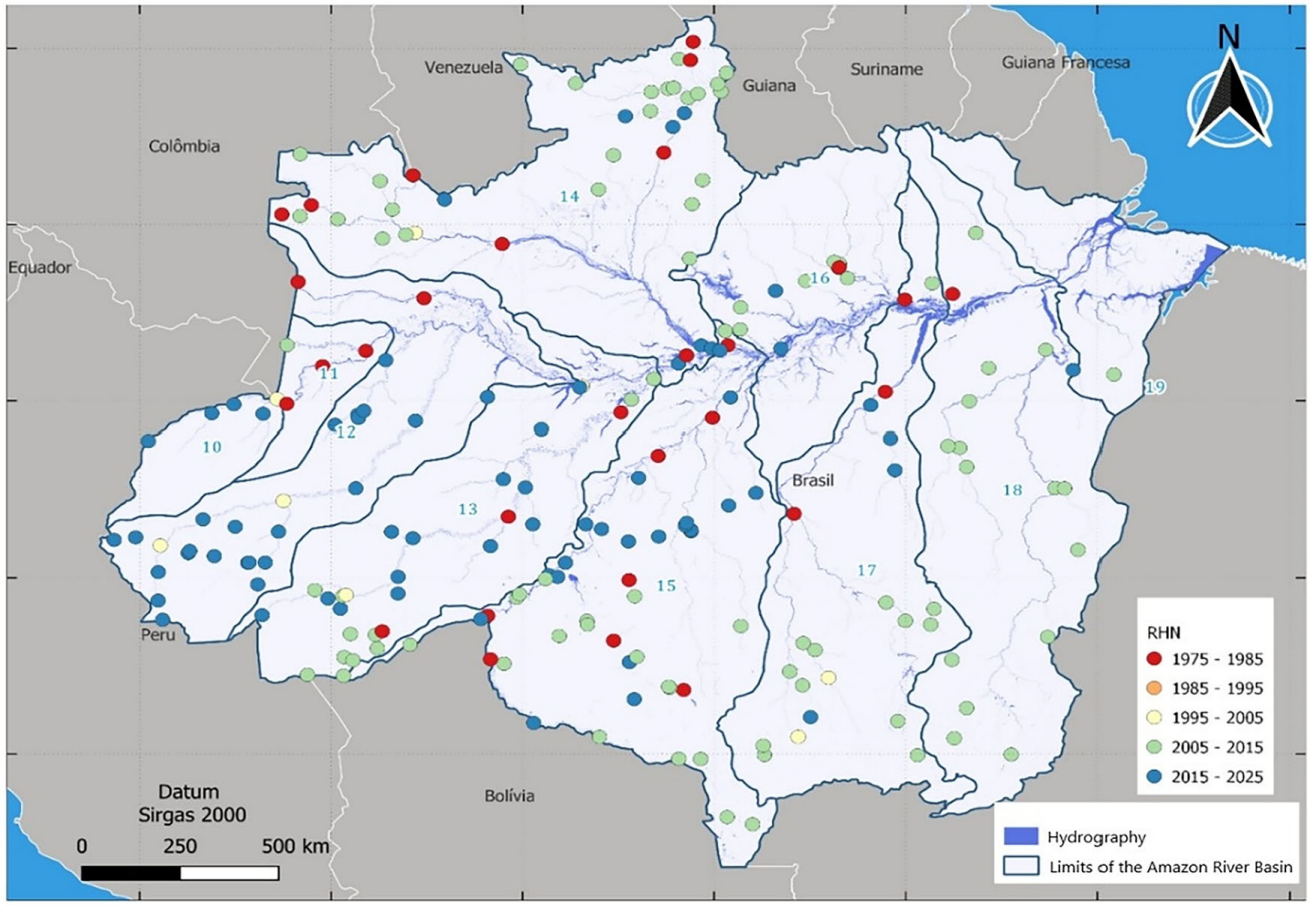
Location of RHN points with progress since the start of operations

Since the use of multiparametric sondes began in the early 2010s, electrical conductivity, pH, turbidity, temperature, and dissolved oxygen have been measured. Figure 4 shows the spatial distribution of RHN monitoring points identified by the range of years in which water quality monitoring began, to illustrate the evolution of monitoring, which totals 206 active measurement points.

From 2005, there has been a visible increase in monitoring, and since 2015, it has been concentrated in the southwestern region and part of the center of the basin, as shown in Fig. 4. Only sub-basin 19 (located at the mouth of the Amazon River) has a single monitoring station, which is the Pacajás station.

In the westernmost part of sub-basin 16 of the Trombetas River, there is low point coverage because it is a region that is difficult to access for obtaining conventional hydrological data. The same is true in the southern part of sub-basin 14 of the Negro River.

**Fig. 5:**
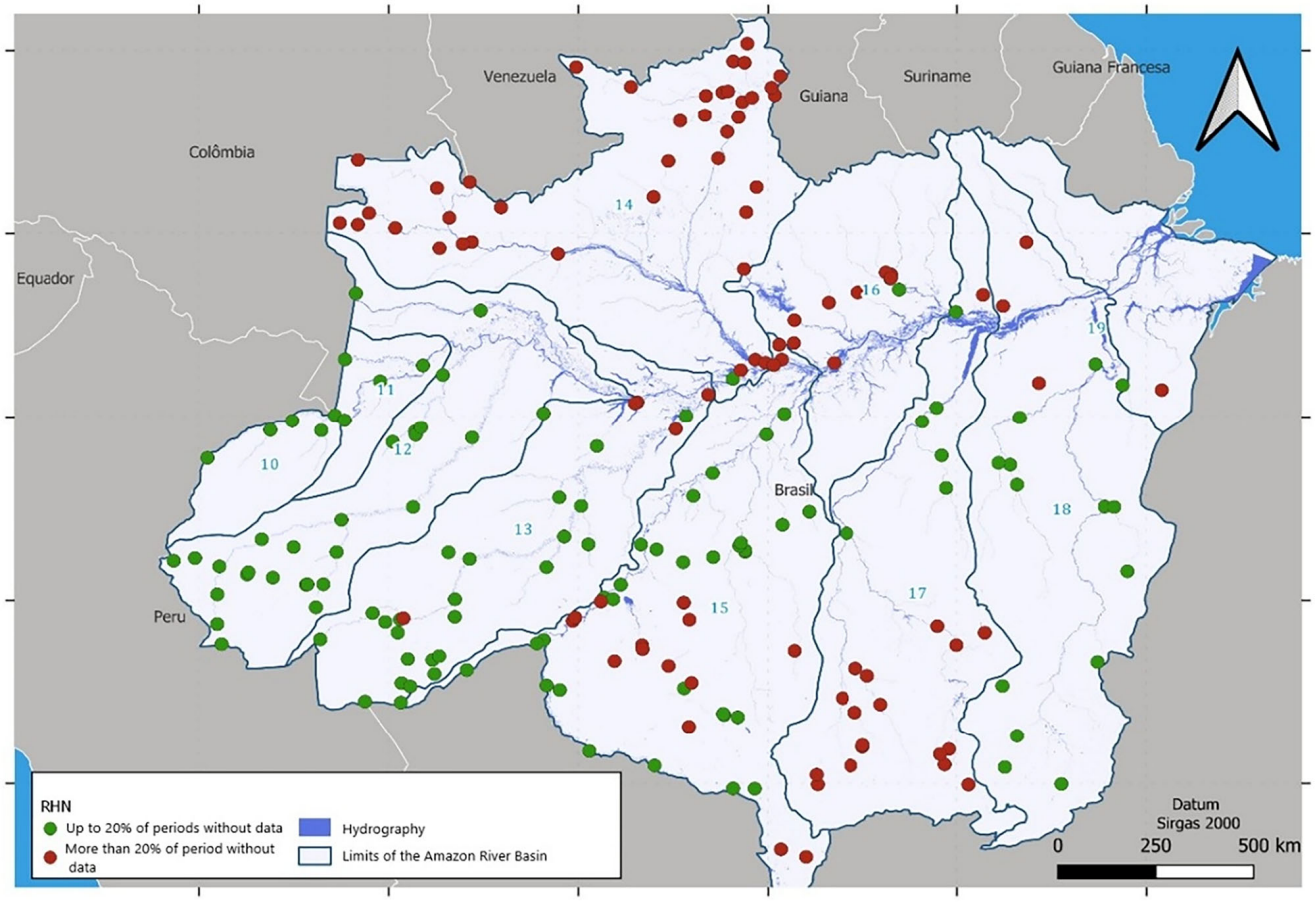
Availability of RHN data

Regarding the availability of information, historical series were analyzed to identify stations with many gaps over time. Figure 5 shows the identification of stations with up to 20% missing data and more than 20% missing data in the series. Periods with many missing values make it difficult to perform statistical analysis of the data; for example, in studies on trends in water quality parameters, the nonparametric Mann-Kendall method requires complete time series, as demonstrated by Samsudin et al. (2017).

There are gaps in data coverage at all stations in the Rio Negro sub-basin and on the left margin of the Amazon River; in the southern parts of the Madeira and Tapajós sub-basins, these gaps account for as much as 50% of the total number of stations. In the west of the basin, the historical series are unbroken, but they are the ones with the most recent start of observation, as seen in Fig. 4. Even with these limitations, the study by Zanin et al. (2024) was able to evaluate the water quality parameters of these stations after a consistency analysis to understand how protected areas tend to improve water quality.

### Surface Water Quality Monitoring Network (RNQA)

The RNQA is coordinated by the states and operated by state environmental agencies. As such, management of the RNQA is decentralized, as defined by the National Water Resources Policy, which emphasizes systematic management without dissociating aspects of quantity and quality (BRAZIL 1997).

According to ANA (2013), the purpose of the RNQA is: “to analyze trends in the evolution of surface water quality, assess whether current quality meets the uses established by the classification of water bodies, identify critical areas in terms of water pollution, assess the effectiveness of management actions to restore water quality, and support planning, concession, licensing, and inspection actions.” The RNQA is also linked to the RNH, so all data measured in the campaigns are made available on the hidroweb to enable systematic management. It can be stated that the main objective of the RNQA is to address gaps and, through the standardization of the network, generate scientific knowledge about water quality in the country.

The procedures for collecting and preserving environmental samples to be used in the RNQA operation must comply with the provisions of the latest edition of the National Guide for the Collection and Preservation of Water, Sediment, Aquatic Communities, and Liquid Effluent Samples, as established by Resolution No. 207 (ANA 2024). Annex II of Resolution No. 903 (ANA 2013), which establishes criteria for the RNQA, presents the twenty-three minimum parameters for water quality monitoring in the RNQA for lotic and lentic environments. The campaigns are conducted twice a year to cover the rainy and less rainy seasons.

For the implementation of the RNQA, the Qualiágua program (ANA 2016) was created, establishing the minimum targets to be met by the states. All states in the study area joined the program, which has two phases of 60 months each. Only the states of Amazonas and Amapá have not yet completed phase 1, and all have joined phase 2. Although the RNQA presents the minimum parameters in Annex II of ANA (2013), in practice, each state has its own capacity to measure the parameters, as shown in Table 1.

**Table 1:** Parameters measured at RNQA monitoring points in the Amazon River basin

State	Qualiágua affiliation	Number of points in 2024	Parameters
Acre	2016	5	CE, T, Turb, OD, pH, DBO, STD, *E. coli*, CT
Amapá	2018	9	CE, T, OD, pH e Turb
Amazonas	2020	54	CE, T, Turb, OD, pH, Cl^-^,Alcal, STD, SST, DBO, DQO, CT, FT, NT
Mato Grosso	2017	32	CE, T, Turb, OD, pH, STD, SST, Alcal, Cl^-^ Total, DBO, DQO, *E. coli*, Orto D, FT, Nit, NA, NT
Rondônia	2016	27	CE, T, Turb, OD, pH, STD, SST, Alcal, Cl^-^ Total, Transp, DBO, DQO, COT, CT, Clorof, Fito, FSR, FT, Nit, NA, NT
Roraima	2016	25	CE, T, Turb, OD, pH, DBO, STD, *E. coli*, CT

*CE* Electrical Conductivity (µS/cm), *T* Water and Air Temperature (°C), *Turb* Turbidity (UNT), *OD* Dissolved Oxygen (mg/L de O_2_), pH, *STD* Total Dissolved Solids (mg/L), *SST* Total Suspended Solids (mg/L), *DBO* Biochemical Oxygen Demand (5 d, 20°C, mg/L de O_2_), *DQO* Chemical Oxygen Demand (mg/L de O_2_), *Cl*^−^ Chlorides (mg/L), *Cl*^*−*^ *Total* Total Chloride (mg/L de Cl), *Alcal* Total Alkalinity (mg/L de CaCO_3_), *Orto D* Dissolved Orthophosphate (mg/L de P), *COT* Total Organic Carbon (mg/L como C), *FT* Total Phosphorus (mg/L de P), *FSR* Reactive Soluble Phosphorus (mg/L de P), *Nit* Nitrate (mg/L de N), *NA* Ammoniacal Nitrogen (mg/L de N), *NT* Total Nitrogen (mg/L de N), *Transp* Water Transparency (m), *Clorof* Chlorophyll α (µg/L), *Fito* Phytoplankton - quantitative and qualitative (number of cells/mL), *E. coli* Escherichia coli (UFC/100 mL), *CT* Total Coliforms (NMP/100 mL)

When analyzing Table 1, it can be inferred that the state of Rondônia is the only state that monitors all the minimum parameters provided for, with Mato Grosso being the second state with the highest number of parameters and measuring the parameter Dissolved Orthophosphate (mg/L of P), which is not provided for in Annex II of ANA (2013). Acre and Roraima monitor the same parameters, while Amazonas is the state that most recently joined the program.

Monitoring sites, which are distributed in coordination with state agencies, can be classified as strategic sites, impact sites, and reference sites in order to eliminate geographical and temporal gaps in monitoring. A strategic point is one located in a border region, at a state boundary, or near large-scale concession projects. Impact points are those in areas affected by potentially polluting human activities. Reference points, on the other hand, are in low-impact environments with the aim of establishing baseline standards, such as in conservation areas or indigenous lands (ANA 2013).

The state with the highest number of points is Amazonas, with 54 measurement points, and the state with the lowest number is Acre (only 5 points). The total number of RNQA monitoring points in the Amazon basin is 152.

**Fig. 6:**
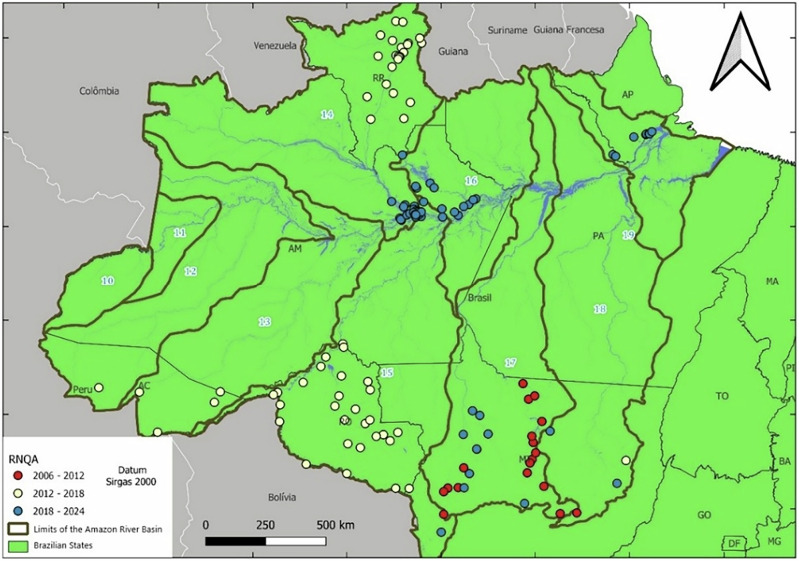
Location of RNQA points with progress since the start of operations

Figure 6 shows the spatial distribution of the data points and the range of years during which each point began operation. Considering the spatial distribution of the data points within state boundaries, the state of Rondônia has the best spatial distribution. When compared with the parameters in Table 1, the state of Rondônia has the highest number of measured water quality parameters. Based on this analysis, it can be inferred that it has the most comprehensive RNQA state network in the study area.

In the state of Acre, the points are concentrated in areas bordering Peru and Bolivia, and in Amapá they are grouped at the mouth of the Amazon River. In the state of Amazonas, the points are distributed at the exutory of sub-basin 14 of the Rio Negro and in sub-basin 16 of the Amazon River between the Madeira and Trombetas rivers. In Mato Grosso, the monitoring points are concentrated in the south-central part of sub-basin 17 of the Tapajós River, with three points in the south of sub-basin 18 of the Xingu River.

Analyzing the Amazon River basin as a whole, the RNQA points are very concentrated in certain areas and need to be expanded in order to achieve the established goal of covering geographical gaps in terms of monitoring density.

In the analysis of historical series, the data is very recent (from 2015 onwards), with the exception of Mato Grosso, which has data measured since 2006, and has few gaps (less than 20%), with most points having a complete series. The RNQA is an evolution, as it provides incentives such as Qualiágua for states that are managing to carry out the operation within their particularities.

### Hidrosat Virtual Network

Hidrosat was created to raise awareness of and systematize the Technical Cooperation Project for Hydrological Space Monitoring of Large Basins, developed by ANA and the French institute IRD (Institut de Recherche pour le Développement); furthermore, it aims to monitor specific parameters and serve as a complement to surface stations. The virtual stations obtained through the use of space sensors embedded in satellites can estimate sediment concentrations, turbidity, and chlorophyll-α, as well as the elevations of virtual stations distributed throughout South America.

Water quality data are obtained from the processing of images from MODIS (MODerate resolution Imaging Spectroradiometer) sensors onboard NASA’s Terra and Aqua satellites for over 20 years, sensors from the Landsat family (TM, ETM+, and OLI), and MSI/Sentinel-2 (Carvalho et al. 2015).

The platform is used for various studies, such as: the assessment of sediment transport carried out by Benatti et al. (2024), in comparative studies between real stations and hydro-sedimentological models that show that they have the same order of magnitude, as done by Silva et al. (2024) in five important Brazilian rivers and in the comparison of sediment key curves with measured data and by Hidrosat, which expands the collection of information where there are no conventional measurements (Condé et al. 2020).

**Fig. 7:**
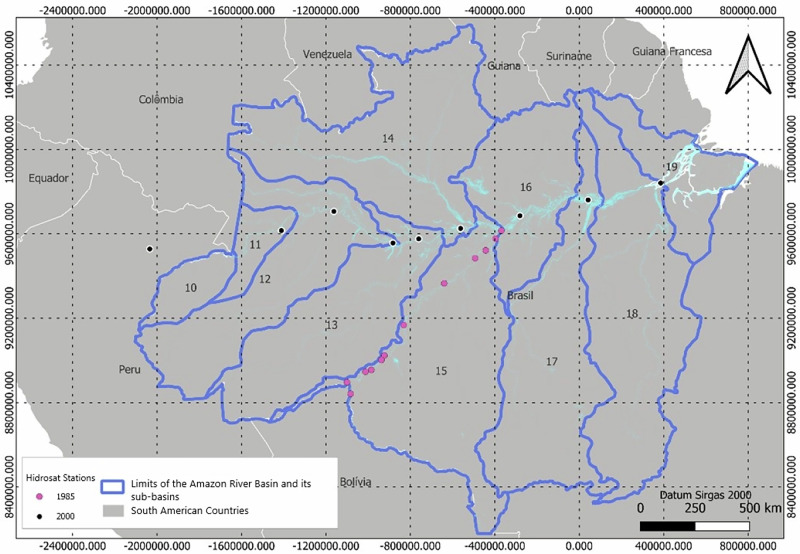
Location of virtual stations measuring suspended sediment (mg/L) from Hidrosat (ANA) and year monitoring began

Regarding water quality information in the study area of this work, only the parameter Suspended Sediment (mg/L) has been measured, distributed along the Amazon River with 8 points in Brazil and datas from 2000 onwards (with one monitoring point in Peru) and distributed along the Madeira River, spatialized across 12 virtual monitoring points since 1985 with Landsat data. The frequency of the data is not well defined due to satellite passes, with reading intervals ranging from 7 to 15 days in the historical series. The spatial distribution of these points and the year monitoring began can be analyzed in Fig. 7.

### Amazonas State Water, Air, and Soil Quality Monitoring Program (ProQAS/AM)

According to Guestrim et al. (2022), ProQAS/AM, created in 2022 and conceived by researchers at UEA, is one of the largest environmental monitoring programs in the world currently in operation, aimed at preserving the Amazon. It has 12 environmental monitoring projects and maintains partnerships with Harvard University, the University of Geneva, and the Max Planck Institute.

ProQAS/AM develops water quality monitoring actions that aim to understand and monitor the conditions of water, soil, and air and scenarios in the face of extreme events (Mamede et al. 2025). The program’s main output is the development of a standardized WQI specifically designed for sewage-carrying rivers in the Amazon. This index was constructed using a robust database containing 342,930 analyses and 161 parameters (collected between 2021 and 2023), designed to reflect local conditions. Unlike the traditional model adopted in Brazil, which was developed by the NSF (National Sanitation Foundation) in the U.S. and adapted by CETESB according to Von Sperling (2014), the WQI formulation proposed by PROQAS/AM is based on calibration criteria for waters with low natural acidity, and its methodology is described by Duvoisin et al. (2025). While the classical method severely penalizes waters with low pH, the index customized by Duvoisin et al. (2025) adjusts the weightings to the Amazonian reality: a pH of 5.0, for example, is classified as normal and ecologically sound for blackwater bodies.

The projects currently being implemented by ProQAS/AM are: Water Quality Monitoring in Greater Manaus, the Madeira River (within the boundaries of the State of Amazonas), the Negro River (between Manaus and São Gabriel da Cachoeira), and the Solimões River (Stage 1: Tefé to Manaus). For the other sections, funding is needed to expand monitoring, such as the Purus, Juruá, Japurá, Solimões (between Tefé and Tabatinga), Amazonas (between Manaus and Parintins), and Frontier and Transboundary Rivers.

**Fig. 8:**
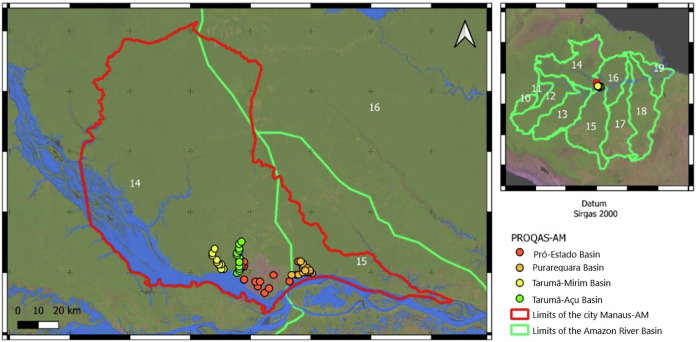
ProQAS/AM systematic monitoring points

To be considered systematic monitoring, parameters must be standardized and measured data must be available. When consulting the program’s website https://www.gp-qat.com/, the freely available data are those from the Greater Manaus Water Quality Monitoring, which is carried out in four basins within the limits of the capital of Amazonas, as shown in Fig. 8, and which totals 55 monitoring points. The other projects mentioned do not yet have water quality data available on their own websites and, therefore, were not spatialized in this study.

The data has been available since 2022 and has already been classified by the WQI developed by Duvoisin et al. (2025) for blackwater rivers and presents the results of the following parameters: Ammoniacal Nitrogen, Total Phosphorus (mg/L), pH, Turbidity (NTU), Dissolved Solids, Thermotolerant Coliforms (NMP/100 mL), Conductivity, Biochemical Oxygen Demand (mg/L), and Dissolved Oxygen (%). In addition, the campaigns are conducted four times a year and the entire database is consistent (regarding the analysis of outliers and periods with missing data), with no missing data for the period from 2022 to 2025. This frequency remains consistent over time, as can be seen in the available data.

### So Hybam

The observation service of geodynamic, hydrological and biogeochemical controls of erosion, So Hybam, has been operating since 2003 and constitutes an international cooperation between research institutions (universities and research centers) and national agencies working in the field of hydrology. The network involves several countries and aims to monitor hydrological, sedimentary and geochemical fluxes in large tropical rivers (Seyler et al. 2022).

The observatory integrates the national hydrological monitoring networks of each participating country and performs measurements of hydrological, hydrogeochemical and suspended matter variables (N’kaya et al. 2020). In this way, it contributes to the understanding of erosion processes, sediment transport and the transfer of materials along river basins.

According to Villar et al. (2012), So Hybam can also be considered a water quality monitoring network, as it allows the evaluation of river discharge and suspended sediment concentrations at the water surface. Since the data are publicly available at the portal https://hybam.obs-mip.fr/ and monitoring is continuous, this initiative can be classified as a systematic monitoring network. The data acquisition intervals may be daily, ten-day, or monthly. The stations are distributed from the Andean headwaters to the main rivers that drain into the Amazon River and track the transport of materials to the Atlantic Ocean, contributing to studies of the global balance of sediments and chemical elements (Cochonneau et al. 2006).

**Fig. 9:**
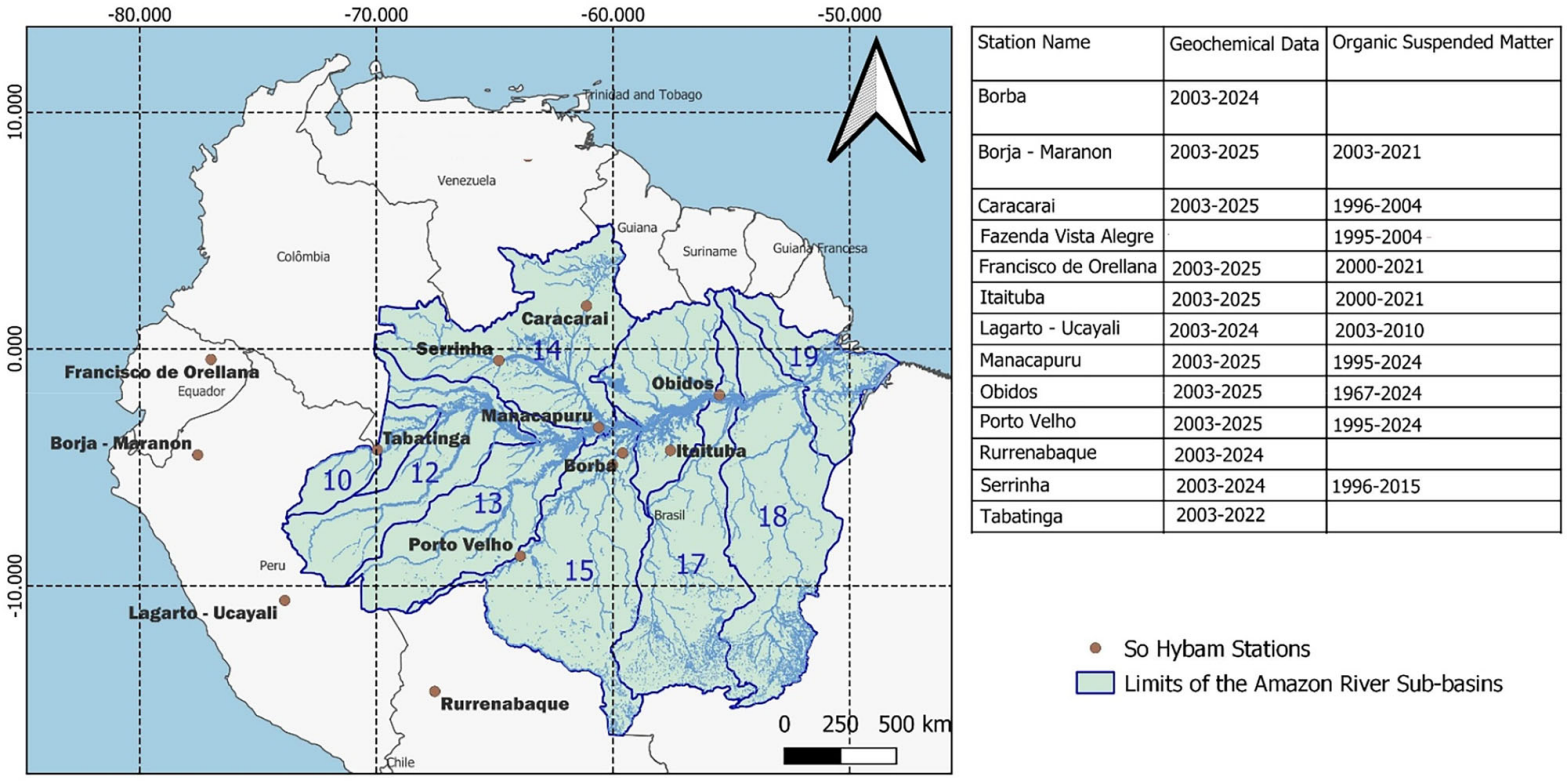
Distribution of the stations of the So Hybam Observatory

The network also includes monitoring in other major tropical basins, such as the Amazon, Orinoco and Congo river basins. In the Amazon basin, 13 monitoring sites were identified (Fig. 9), with available geochemical and water quality data. The monitored parameters include suspended matter concentration (mg/L), water temperature, electrical conductivity and pH.

In addition, hydrological variables such as water level and discharge are recorded, the latter being obtained through the development of rating curves. The description of the parameters, sampling frequency and instrumentation is presented in Table 2.

**Table 2:** Description of the variables measured by the So Hybam Observatory

Parameter		Frequency	Instrument/method	Observation
Hydrological variables	Water level	Daily	Staff gauges/limnigraphs	Operated by national agencies
	Discharge	Daily	ADCP	
Physicochemical parameters	Water temperature	10-day/monthly	Thermo-conductivity meter	Sampling every 10 days with 1000 mL samples sent to partner laboratories
	Electrical conductivity	10-day/monthly	Thermo-conductivity meter	
	pH	10-day/monthly	pH meter	
	Suspend organic matter	10-day/monthly	Surface sampling	
Geochemical variables	Major ions (Ca, Mg, K, Na, HCO₃, Cl, SO₄, Fe, Al, Si, Sr, NO₃, F, PO₄)	Monthly	HPLC, IC, ICP-AES	Collected and filtered in the field, stored in different containers, and sent to partner laboratories
	Trace and rare elements (Ti, V, Cr, Mn, Co, Ni, Cu, Zn, As, Rb, Zr, Mo, Cd, Ba, U, La, Ce, Pr, Nd, Sm, Eu, Gd, Tb, Dy, Ho, Er, Yb, Lu, Tm)	Monthly	ICP-MS	
	Silica	Monthly	ICP-AES	
	Carbon (DOC, POC)	Monthly	Infrared Spectroscopy	
	Isotopes	Quartely	TIMS, MS	

Adapted from Cochonneau et al. (2006)

*Ca* Calcium, *Mg* Magnesium, *K* Potassium, *Na* Sodium, *HCO*_3_ Bicarbonate, *Cl* Chloride, *SO*_4_ Sulfate, *Fe* Iron, *Al* Aluminium, *Si* Silicon, *Sr* Strontium, *NO*_3_ Nitrate, F Fluoride, *PO*_4_, Phosphate, *Ti* Titanium, *V* Vanadium, *Cr* Chromium, *Mn* Manganese, *Co* Cobalt, *Ni* Nickel, *Cu* Copper, *Zn* Zinc, *As* Arsenic, *Rb* Rubidium, *Zr* Zirconium, *Mo* Molybdemum, *Cd* Cadmium, *Ba* Barium, *U* Uranium, *La* Lanthanum, *Ce* Cerium, *Pr* Praseodymium, *Nd* Neodymium, *Sm* Samarium, *Eu* Europium, *Gd* Gadolinium, *Tb* Terbium, *Dy* Dysprosium, *Ho* Holmium, *Er* Erbium, *Yb* Ytterbium, *Lu* Lutetium, *Tm* Thulium, *DOC* Dissolved Organic Carbon, *POC* Particulate Organic Carbon, *HPLC* High-Performance Liquid Chromatography, *IC* Ion Chromatography, *ICP-AES* Inductively Coupled Plasma Atomic Emission Spectroscopy, *ICP-MS* Inductively Coupled Plasma Mass Spectrometry, *TIMS* Thermal Ionization Mass Spectrometry, *MS* Mass Spectrometry, *ADCP* Acoustic Doppler Current Profiler

Regarding data periodicity, Fig. 9 presents the years of available monitoring data. The longest historical series corresponds to the Óbidos station, with more than 50 years of observations, while the remaining series mainly begin in the 2000s. This availability of data allows the evaluation of hydrological and geochemical trends.

Regarding the spatial distribution of stations in the Amazon basin (Fig. 9), a concentration can be observed in the Andean regions (where the headwaters of the Amazon River are formed), along the main channel of the Amazon River up to the Óbidos station, and in important sub-basins that contribute to the main system. These include the Serrinha and Caracaraí stations, located in the Negro River sub-basin, and the Porto Velho station on the Madeira River.

This spatial distribution is fundamental to achieving the objective of the So Hybam observation network, which is to understand the dynamics of sediment.

### Overview of Systematic Water Quality Monitoring

**Fig. 10:**
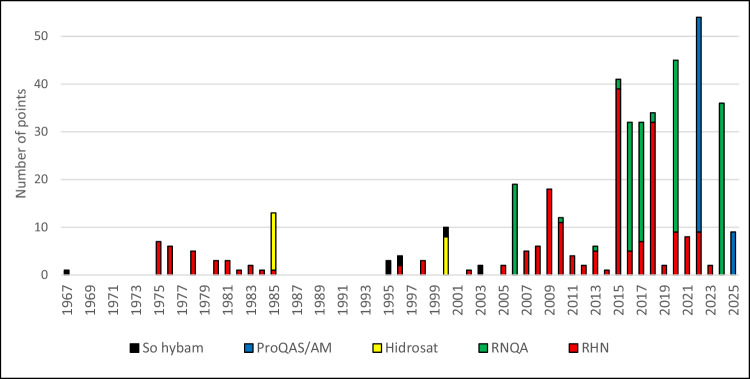
Number of monitoring points initiated over time by the RHN, Hidrosat, RNQA and ProQAS/AM systematic monitoring networks in the Amazon River basin

Knowledge of water quality data is extremely important for water management, as this study shows that the five networks presented have your characteristics. Figure 10 shows the evolution of the start year of each monitoring point of the five networks over time, where it is possible to highlight the expansion of monitoring over the years. With this it is possible to confirm the importance of RHN as a pioneer in systematically obtaining water quality data in the Amazon River basin, with the first stations in the 1970s, and as the network with the largest number of measurement points and with homogeneous distribution throughout the study area.

The period from 1986 to 1995 (Fig. 10) is marked by the absence of any new monitoring stations, and when analyzing the historical series of the RHN, there are periods with missing data at many stations (analyzed in Fig. 5). Until 2005, there were also few new stations, but the scenario changed in 2015, when the RNQA began operating and maintained a growing number of new points in 2016, 2017, 2018, 2019, and 2023 (Fig. 10). The year 2022 is marked by the start of systematic monitoring by ProQAS/AM, with 55 points added this year. This shows that there is a history of monitoring, but that it has been intensified since the creation of the RNQA.

The So Hybam covers the international portion of the Amazon River basin and is the only monitoring network that characterizes the basin’s hydrogeochemistry. This information is essential for identifying dissolved minerals in the water, which are important for the characterization of water bodies, such as in the classification proposed by Sioli (1984), which categorizes Amazonian rivers into white-water, black-water, and clear-water types

**Fig. 11:**
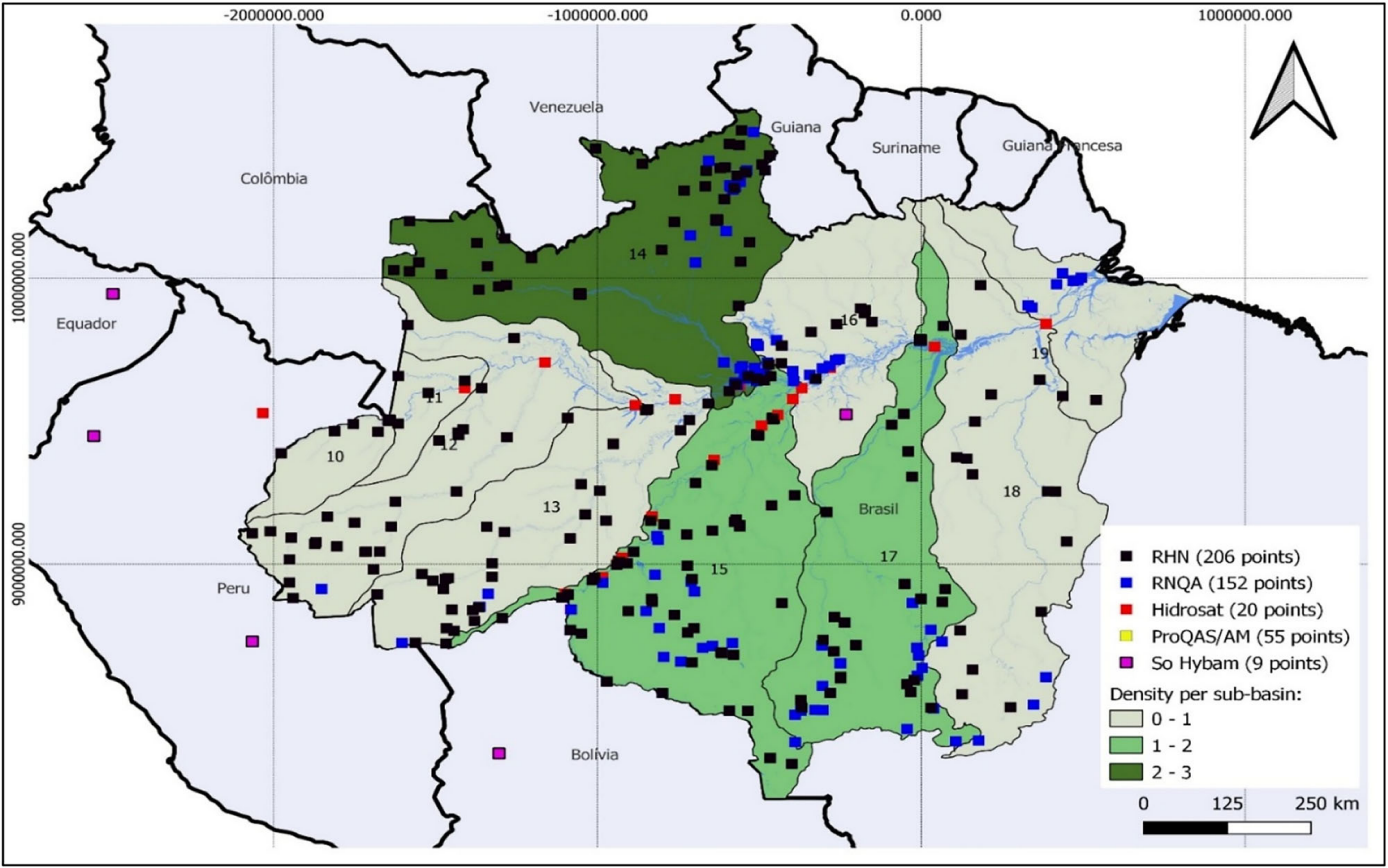
Location and density of RHN, RNQA, Hidrosat, ProQAS/AM, and So Hybam points

The total number of points up to the year 2024 that are located within the boundaries of the study area and correspond to the Brazilian portion of the basin is 442 (RHN: 206 points, RNQA: 152 points, Hidrosat: 20 points, ProQAS/AM: 55 points and So Hybam: 9 points), all of which are specialized in Fig. 11. As for the density of the points, the analysis is carried out in accordance with the recommendation of Resolution No. 903 (ANA 2013), which, for the states of Region 1, is 1 point per 10,000 km², which in this case considers the area of each sub-basin. Each density per sub-basin is shown in Fig. 11.

Sub-basins 14 Negro River, 15 Madeira River and 17 Tapajós River are the only ones that meet the recommendation. However, when analyzing spatial distribution, in the Rio Negro sub-basin the points are concentrated in the southern region and at the outlet, while in the center there is no coverage. This concentration in certain areas also occurs in other sub-basins, such as at the headwaters of sub-basin 16. The Xingu and Paru sub-basin 18 have the lowest density.

Thus, the assessment should not be based solely on density, as this can “mask” the information, as occurs in sub-basin 14, which meets the recommendation, but where the points are concentrated in certain areas. In addition, the design of the water quality network has several methodologies that began in the 1960s (Sanders et al. 1983; Sharp 1971) and, according to Cruz (2024), there is still no universally accepted methodology for network design, and it is common to find networks designed arbitrarily and without methodological criteria. Therefore, more robust research is needed to assess whether the current configuration in Fig. 11 is the most efficient, as monitoring should be sought that is not costly, such as redundant monitoring points or those with excessive parameters.

Systematic information exists within the study area and can be used for numerous studies and to establish standards. However, the data are limited to a few parameters in the oldest network with the best spatial coverage, which is the case of the RHN, or have many analysis parameters but do not cover the entire study area (RNQA and ProQAS/AM).

**Fig. 12:**
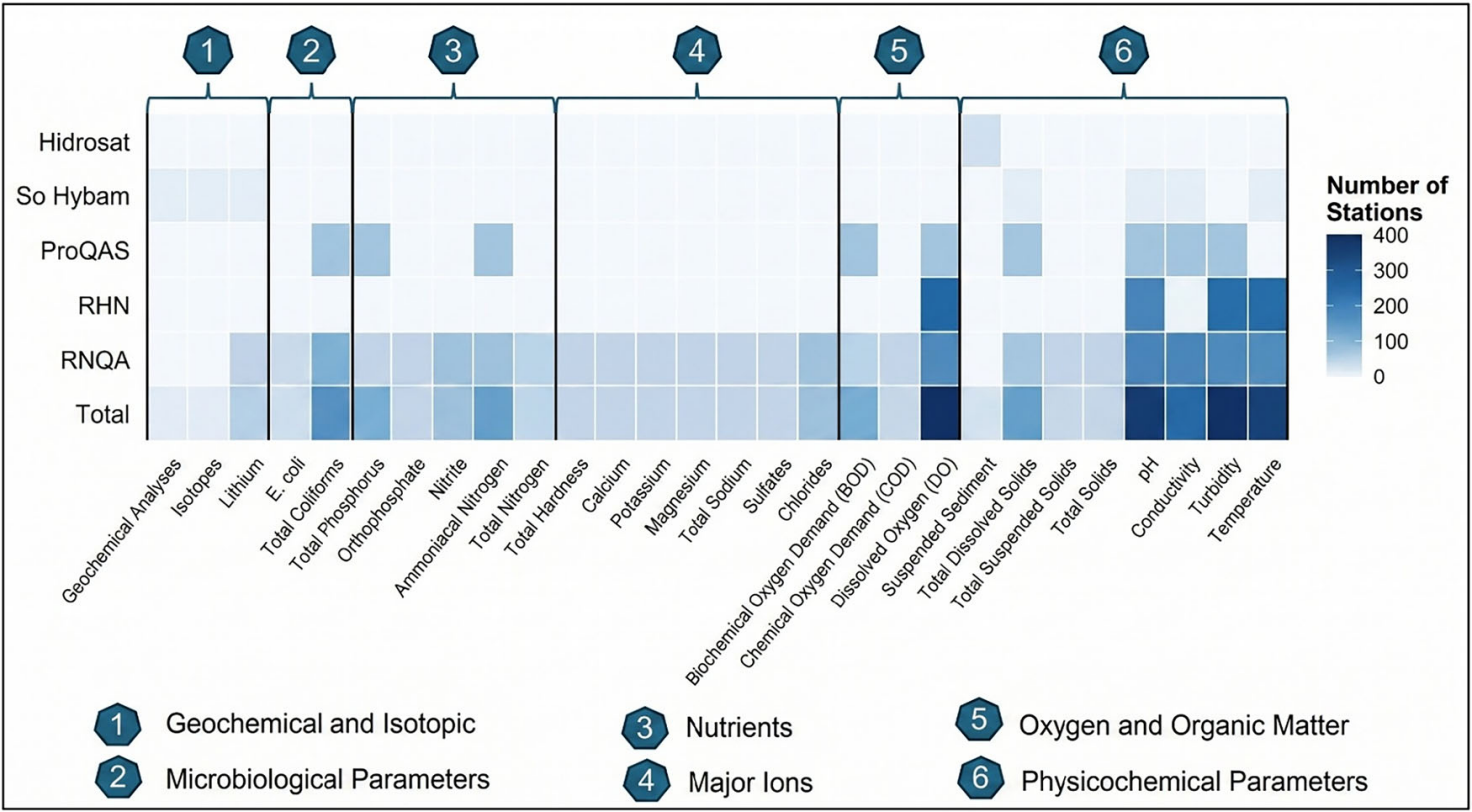
Heatmap showing the assessment of water quality parameters by monitoring point

In addition to the spatial and temporal analyses of the monitoring stations, an evaluation of the parameters measured at each monitoring point and a classification of the network types were conducted, as shown in the heat map in Fig. 12. The parameters are also grouped into geochemical and isotopes, microbiological, nutrients, ions, oxygen and organic matter, and physicochemical.

This summary highlights the main differences between the monitoring initiatives and provides an overview of the types of data available in each database. The Hidrosat network focuses exclusively on estimating suspended sediments from satellite data. The RHN measures only basic water quality parameters. The RNQA and ProQAS/AM networks have similar monitoring approaches, covering basic parameters and nutrient-related variables. In contrast, the So Hybam Observatory provides comprehensive geochemical information, although it has a more limited focus on nutrient monitoring.

Figure 12 shows that the water quality parameters with the highest number of measured stations (more than 300) are Dissolved Oxygen (DO), pH, Turbidity, and Temperature. Future research on the Amazon River and decision-making by public managers are limited to these parameters when considering the basin as a whole.

## Discussion

The monitoring networks presented are well-established and serve as a basis for decision-making, in addition to contributing to the improvement of the monitoring infrastructure in the Amazon River basin, as exemplified by the studies conducted by Duvoisin et al. (2025) to establish an IQA on the Rio Negro and by Zanin et al. (2024) to assess water quality on indigenous lands. However, when analyzing the context of southeastern Brazil and other countries, it is observed that systematic water quality monitoring in the Amazon region is not yet consolidated, as initiatives are still recent and do not cover the entire region, as shown in the results of the RNQA and ProQAS/AM.

European Union countries, such as the United Kingdom, have over 150 years of water quality monitoring on the River Thames, which allows for the assessment of how environmental indicators have changed over time. The Thames River monitoring network is considered the oldest in the world, according to the study by Peter Jarvie et al. (2025), with measurements of parameters such as chloride and biochemical oxygen demand having been conducted since 1868. This century-long history stands in stark contrast to the Amazon Basin, where the lack of long-term historical data series prevents a deep understanding of trends in anthropogenic degradation and climate change.

In the United States, the U.S. Geological Survey (USGS) and the Environmental Protection Agency (EPA) jointly developed the Water Quality Portal (WQP). The WQP integrates water quality data that was previously decentralized and collected by various institutions, often in non-standardized formats. The system establishes a data standard and aims to provide a single point of access for water quality information. Currently, the portal compiles records from the late 19th century to the present day (Read et al. 2017). In Brazil, the RNQA has a similar objective to that of the WQP in the United States, seeking to integrate information from different monitoring initiatives. However, as noted earlier, the data available for the Amazon River basin are still relatively recent.

Furthermore, while the U.S. WQP has successfully achieved compatibility among its databases, the Amazonian context still suffers from poor data harmonization. The five networks operating in the region function independently, without a unified protocol to ensure that analytical methods and laboratory limits for parameters are equivalent, which hinders the creation of an integrated database. To overcome this limitation, it is critical and technically feasible to design a structure that systematically integrates these five monitoring networks and other initiatives that may emerge over the years, such as the availability of non-systematic data from other.

The study by Lin et al. (2024) also highlights limitations in the Chinese context, emphasizing the lack of a publicly accessible water quality dataset covering the entire country. In response to this limitation, the authors developed a public water quality data repository by integrating three pre-existing, hard-to-access datasets covering the period from 1980 to 2022. An important global initiative is the Global River Water Quality Archive (GRQA), developed by Virro et al. (2021), which aims to integrate national, continental, and global datasets. The archive includes more than 17 million measurements collected between 1898 and 2020 and is publicly available. The data cover various regions of the world, including the United States and Canada, Brazil and Chile, South Africa, Oceania, East Asia, and Western Europe.

Focusing on the Amazon region, the Amazon Cooperation Treaty Organization (OTCA) established the Amazon Regional Observatory (ORA) in 2021, which promotes initiatives aimed at integrating various types of monitoring, including water, forests, wildfires, health, public safety, and indigenous peoples (OTCA, 2023). In this context, the Redes ORA repository compiles data from Brazil, Bolivia, Ecuador, Peru, and Colombia. The system integrates the Amazon Hydrological Network (with hydrological data provided by national agencies), the monitoring stations of the So Hybam Observatory, and the Water Quality Network, which in Brazil corresponds to data provided by the RNQA, comprising 40 stations, in addition to four stations operated by Bolivia. Although ORA represents a major institutional advance for transboundary water governance, its actual scientific utility is severely limited by the low spatial density of monitoring points and the incompatibility of sampling frequencies among countries, highlighting that international political integration has advanced faster than technical and methodological synchronization.

When comparing it to other water quality monitoring networks in Brazil, the Automatic Surface Water Quality Monitoring Network operated by CETESB in the state of São Paulo stands out. This network began operating in 1998 and takes measurements every five minutes at 31 monitoring stations, with precipitation records also available at 14 of these stations since 2019. CETESB also provides data through the INFOÁGUAS platform, which includes information on 26 water quality parameters with records dating back to 1974. Also in southeastern Brazil, the monitoring network operated by the Minas Gerais Water Management Institute (IGAM) stands out, conducting systematic monitoring since 1997 in the state’s river basins, with data available on its own platform. In addition, the HidroWeb system of the National Water and Basic Sanitation Agency (ANA) includes 842 monitoring stations with water quality data operated by IGAM, covering the São Francisco, Leste, and Paraná river basins.

This discrepancy between the Southeast and North regions of Brazil highlights a profound regional imbalance in public policy and environmental investment. While the state of São Paulo uses high-frequency, real-time automatic telemetry to monitor small-scale watersheds, the Amazon Basin relies on manual, seasonal, and low-frequency sampling, which is concerning given the impacts caused by extreme events such as frequent droughts.

One of the main focuses of this study is to establish detailed knowledge regarding the systematic acquisition of water quality data in the Brazilian portion of the Amazon River basin. The results indicate that each monitoring network has unique characteristics regarding the number of parameters observed and the presence of gaps in the data series. However, the research also highlights recent initiatives, such as the RNQA and the ProQAS/AM program, which include a greater number of monitored parameters and greater potential to effectively characterize the dynamics of these watercourses, which are of global relevance.

The integration, consolidation, and public availability of environmental data represent a global trend, not only for water quality parameters but for environmental monitoring in general. Based on the results obtained in this study and the consolidation of the available datasets summarized in Fig. 11, it is possible to conduct unprecedented studies on the current state of water quality in the Amazon River basin. Currently, many existing studies rely on non-systematic monitoring networks that lack continuous historical time series. In this sense, the present study represents an initial step toward the development of an integrated database that will allow for the characterization of the different watercourses in the Amazon basin, as well as the assessment of trends in water quality parameters using the longest available observation records. Furthermore, it opens opportunities for studies that integrate water quality monitoring with land use and land cover variables, as well as other hydrometeorological variables.

## Conclusion

The study allowed for an in-depth analysis of existing data on surface water quality in one of the world’s most important rivers. Understanding water quality is essential for complying with environmental guidelines and ensuring the safety of the many users of water resources. Based on this research, it was possible to characterize the frequency, parameters, and periodicity of each of the systematic monitoring networks on the Amazon River.

Continuous, standardized monitoring with a wide range of available data is essential for understanding the dynamics of water bodies and their regional characteristics. The study demonstrated that the creation of a specific water quality network is a recent development in the Amazon, driven by the establishment of the RNQA, and reinforces the need to support initiatives such as ProQAS/AM, as well as the consolidation of the SO Hybam network, which conducts state-of-the-art monitoring through international partnerships. The data generated by the RHN proved to be the most widely disseminated and frequent, although they have limitations regarding the number of physicochemical and biological variables assessed.

However, gaps were found regarding the monitored parameters and the spatial coverage necessary to fully achieve the monitoring objectives. Furthermore, the temporal discontinuities identified, especially in the RHN, compromise the consistency and robust analysis of historical water quality time series in the region, which undermines environmental monitoring in remote areas.

Global trends point to a recent trend toward system integration to facilitate public access to environmental data. In the Amazon, this demand is beginning to become a reality, and this article marks the first step toward the consolidation of water quality data in a region of vast socio-environmental importance. The integration of all these platforms is essential for advancing monitoring in the Amazon Basin, serving as a basis for academic research focused on the impacts of extreme weather events and anthropogenic pressures, as well as for the proper classification and categorization of Amazonian water bodies.

## Data Availability

All data in the article are available free of charge on the hidroweb portal: https://www.snirh.gov.br/hidroweb/apresentacao and on the website: https://www.gp-qat.com/proqas.
